# Improving Hepatocellular Carcinoma Surveillance in Ambulatory Hepatology: A PDSA Quality Improvement Initiative

**DOI:** 10.3390/nursrep16060188

**Published:** 2026-05-29

**Authors:** Anjana Mary Jacob, Satheesh Nair, Drew A. Wells, Beatrice Bailey, M. Dennis Leo

**Affiliations:** 1 Transplant Hepatology, Methodist University Hospital Transplant Institute, Memphis, TN 38103, USA; 2 Methodist University Hospital Transplant Institute, University of Tennessee Health Science Center, Memphis, TN 38163, USA; 3 Internal Medicine, Department of Pharmacy, Methodist University Hospital, Memphis, TN 38104, USA; 4 College of Nursing, Arkansas State University, Jonesboro, AR 72467, USA; 5 College of Pharmacy, University of Tennessee Health Science Center, Memphis, TN 38163, USA

**Keywords:** hepatocellular carcinoma surveillance, quality improvement, healthcare personnel education, PDSA cycle, guideline adherence

## Abstract

**Background:** Hepatocellular carcinoma (HCC) is the third leading cause of cancer-related mortality worldwide. Despite established guidelines recommending semiannual surveillance for high-risk patients, real-world adherence remains inconsistent. Gaps in healthcare personnel knowledge and care coordination are recognized contributors to this implementation failure. **Methods:** Under IRB review, a quality improvement project using the Plan-Do-Study-Act (PDSA) framework was conducted in an ambulatory liver clinic in the southwestern United States. One PDSA cycle was completed. Retrospective and prospective chart reviews (n = 50 charts each) were conducted to assess HCC surveillance ordering and scheduling rates. Thirty healthcare personnel completed investigator-developed pre- and postintervention surveys measuring knowledge and perceptions. **Intervention:** A structured educational session grounded in current American Association for the Study of Liver Diseases (AASLD) surveillance guidelines was delivered to the full interdisciplinary clinic team, incorporating clinic-specific compliance data and role-specific coordination responsibilities. Results: Knowledge scores improved significantly from a mean of 41.67% to 95.33% (t [29] = −20.27, *p* < 0.001, d = 3.70). Perception scores improved (Wilcoxon z = −4.30, *p* < 0.001). Surveillance ordering increased from 88% to 94% and scheduling from 60% to 80%. **Conclusions:** A single structured educational PDSA cycle was associated with significant improvements in healthcare personnel knowledge and perceptions and with improved ordering and scheduling of HCC surveillance imaging. Postintervention imaging completion and result review rates were not assessed within the available follow-up period. Run chart monitoring of surveillance metrics across subsequent PDSA cycles is planned to evaluate sustainment and guide iterative improvement.

## 1. Introduction

Hepatocellular carcinoma (HCC) is the most common primary liver malignancy and ranks third among causes of cancer-related mortality globally, accounting for approximately 800,000 deaths annually [1]. Over the past four decades, liver cancer incidence has nearly tripled and mortality has doubled in the United States, trends closely paralleling the rising prevalence of chronic liver disease, the principal substrate from which HCC develops [2]. Approximately 80% of HCC cases arise in individuals with underlying cirrhosis, and the five-year survival rate for HCC remains approximately 22% overall, falling below 10% when the disease is detected at symptomatic or advanced stages [3,4]. This poor prognosis reflects the critical importance of early detection, as patients diagnosed with early-stage disease have substantially greater access to curative therapies, including liver transplantation, surgical resection, and locoregional ablation [5].

The American Association for the Study of Liver Diseases (AASLD) recommends semiannual surveillance with abdominal ultrasound with or without serum alpha-fetoprotein (AFP) measurement for all patients with cirrhosis and for those with chronic hepatitis B infection who meet specific risk criteria [6,7]. The evidence base supporting this recommendation is well established: a landmark prospective randomized study demonstrated that systematic semi-annual surveillance reduced HCC-related mortality by approximately 37% in a high-risk cohort with chronic hepatitis infection, primarily by enabling earlier, more treatable diagnoses [8]. Despite this evidence, adherence to surveillance in routine clinical practice remains alarmingly poor. A multicenter retrospective cohort study found that more than 60% of patients newly diagnosed with HCC had not received recommended surveillance imaging in the period preceding their diagnosis [9], underscoring the persistent and consequential gap between guideline-directed care and real-world implementation.

Multiple factors contribute to suboptimal surveillance adherence. System-level barriers, including fragmented care coordination, competing clinical priorities, and limited infrastructure for population-level tracking of surveillance status, have been consistently identified as drivers of low adherence rates. At the provider level, variable awareness of surveillance guidelines, uncertainty about which patients qualify as high-risk, and unclear delineation of roles within multidisciplinary teams further compromise the consistent implementation of these guidelines. In ambulatory hepatology settings in particular, effective HCC surveillance depends on coordinated action across a range of roles. Clinicians must identify eligible patients and place orders, nurses and medical assistants must coordinate scheduling and patient communication, and administrative staff must ensure imaging is completed and results are accessible for review within recommended timeframes. A knowledge gap or care coordination failure at any point along this chain can result in missed or delayed surveillance, with potentially life-altering consequences for individual patients [10].

Evidence-based practice supports targeted educational interventions as a viable strategy to address provider knowledge deficits and improve adherence to clinical guidelines. Studies examining educational approaches in the context of cancer screening and chronic disease surveillance have demonstrated improvements in provider knowledge, attitudes, and practice behaviors following structured training, particularly when education is tailored to the specific practice context, incorporates local performance data, and engages the full interdisciplinary team rather than clinicians alone [10]. The PDSA cycle provides an iterative framework for testing, evaluating, and refining such interventions in real-world clinical settings [11], making it well-suited to the complexity and interdependence of surveillance workflows in ambulatory practice.

**Figure 1:**
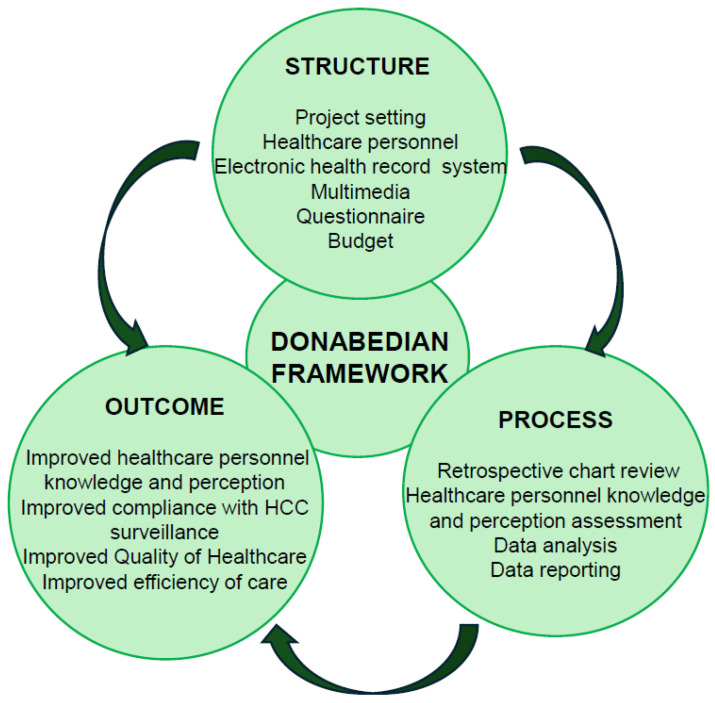
Theoretical framework: Donabedian model applied to HCC surveillance quality improvement. The Donabedian model organized evaluation across three domains: structure (project setting, healthcare personnel, EHR system, and supporting resources), process (chart review, knowledge and perception assessment, and data analysis), and outcome (improved personnel knowledge, HCC surveillance compliance, and quality of care). These domains guided a structured educational intervention implemented within a single PDSA cycle, with iterative cycles and longitudinal monitoring planned for subsequent quarters.

This quality improvement (QI) project was conducted in an ambulatory liver clinic within a transplant institute in the Southwestern United States. The clinic serves patients with cirrhosis of varied etiologies, chronic hepatitis B, and post-liver transplant status, populations at elevated risk for HCC who require consistent surveillance monitoring. The project applied the PDSA framework, guided by the Donabedian model (Figure 1) of quality evaluation, to implement and evaluate a structured educational intervention targeting healthcare personnel knowledge and perceptions as proximate drivers of surveillance adherence. This report describes one completed PDSA cycle and lays the foundation for iterative cycles with longitudinal outcome monitoring planned for subsequent quarters.

## 2. Materials and Methods

### 2.1. Evidence Base and Theoretical Framework

A focused review of the peer-reviewed literature informed the intervention’s content and design. Sources included current AASLD clinical practice guidelines, systematic reviews, and cohort studies examining HCC surveillance adherence, as well as studies evaluating educational interventions targeting provider knowledge and surveillance utilization [1,9,10]. The evidence consistently identified modifiable provider-level factors, including knowledge deficits, attitudinal barriers, and role ambiguity, as contributors to suboptimal adherence, supporting structured team education as a targeted, evidence-based intervention strategy.

The Donabedian framework, comprising structure, process, and outcome, guided project design and evaluation. Structural inputs included the composition and training of the interdisciplinary clinic team, as well as the educational session itself. Process measures included HCC surveillance ordering and scheduling rates as indicators of guideline-concordant workflow. Outcome measures included healthcare personnel knowledge and perception scores as proximate indicators of the behavioral and attitudinal changes expected to drive process improvement. The PDSA model [11] operationalized the improvement cycle: the Plan phase identified the practice gap through baseline chart review and literature synthesis; the Do phase implemented the educational intervention; the Study phase analyzed pre- and post-survey and chart review data; and the Act phase generated recommendations to inform subsequent iterative cycles.

### 2.2. Setting and Participants

The project was conducted in an ambulatory liver clinic within the transplant institute of an academic hospital in the southwestern United States. The clinic provides specialized hepatology services, including pre- and post-liver transplant evaluations and management of chronic liver disease, and is supported by an interdisciplinary care team and an electronic health record system.

**Figure 2:**
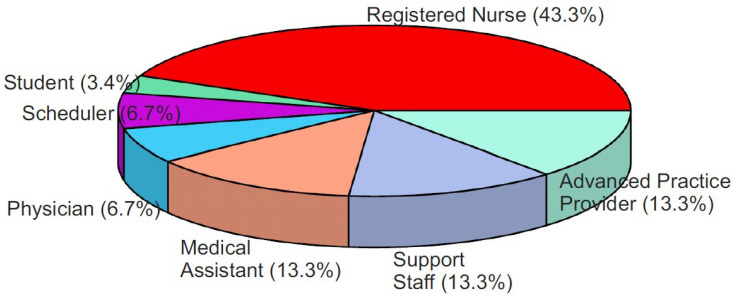
Clinical role distribution of healthcare personnel participants (N = 30). Values represent the percentage of total participants and the corresponding count (n) for each role category. Registered nurses comprised the largest group (43.3%, n = 13), followed by advanced practice providers, medical assistants, and support staff (13.3% each, n = 4 each). Physicians and schedulers each represented 6.7% (n = 2), and one student participant represented 3.4% of the sample.

Healthcare personnel participants (N = 30 of 40 eligible; 75% participation rate) represented all roles involved in HCC surveillance coordination: physicians, advanced practice providers, registered nurses, medical assistants, schedulers, support staff, and students (Figure 2). Inclusion criteria required employment in the clinic during the implementation period, direct involvement in the care of patients at risk for HCC, and a minimum of one year of ambulatory clinical experience. Personnel absent on the day of the educational session were excluded.

Chart review included adult patients with cirrhosis of any etiology, chronic hepatitis B infection with or without cirrhosis, or post-liver transplant status conferring HCC risk (n = 50 preintervention; n = 50 postintervention). Post-liver transplant patients were included because recipients without prior HCC who met institutional protocol criteria remain at risk due to ongoing immunosuppression and underlying liver disease etiology; surveillance indications were confirmed consistent with institutional protocol and AASLD guidance. Patients with active HCC undergoing treatment, those currently in transplant evaluation, and those listed for liver transplantation were excluded. The preintervention chart review covered patient records from calendar year 2023, and the postintervention chart review covered records from the three months following the educational session. Charts were selected consecutively from the clinic’s scheduling records within each review window. No patient appeared in both samples, as the review periods were distinct and non-overlapping. A chart review was performed by the principal investigator using a standardized abstraction form. A second reviewer independently reviewed a 10% random sample of charts to assess inter-rater reliability; agreement exceeded 95% for all surveillance indicators. Reviewers were not blinded to the intervention period, which is acknowledged as a potential source of observation bias.

### 2.3. PDSA Cycle and Intervention

Plan: Baseline HCC surveillance adherence was assessed through retrospective chart review of 2023 patient records. Preintervention surveys established healthcare personnel’s knowledge and perceptions as the modifiable targets for the intervention. The literature synthesis confirmed structured team education as an evidence-based strategy aligned with the identified gaps.

Do: A single structured educational session, approximately 60 min in duration, was delivered in person as an interactive presentation to the assembled interdisciplinary team. The session was delivered by the principal investigator (a doctoral-prepared nurse practitioner) to all available clinic staff simultaneously. Attendance was voluntary; personnel absent on the day of the session were excluded from the study. Content, grounded in current AASLD guidelines, addressed the clinical rationale for semiannual HCC surveillance, eligibility criteria for surveillance (including cirrhosis etiology and hepatitis B risk stratification), recommended imaging modalities and AFP use, the consequences of missed or delayed screening, clinic-specific surveillance compliance data, and the distinct coordination role of each team member, from eligibility identification and order placement through scheduling, patient contact, and result review. The session incorporated role-specific case scenarios to contextualize responsibilities within the clinic workflow. No formal algorithms, checklists, or electronic reminders were implemented as part of this cycle, though these are planned for subsequent cycles. No follow-up reinforcement sessions were delivered within the timeframe of this cycle. The session was scheduled in collaboration with clinic leadership to minimize disruption to patient care.

Study: Outcomes were evaluated across three data streams: (1) pre- and postintervention knowledge questionnaire scores; (2) pre- and postintervention Likert-scale perception scores; and (3) prospective chart review surveillance ordering and scheduling rates. The knowledge instrument was a 15-item multiple-choice questionnaire assessing familiarity with current AASLD HCC surveillance guidelines, including eligibility criteria, recommended imaging modalities, surveillance intervals, and AFP use; scores were calculated as the percentage of correct responses (range 0–100%). The perception instrument was 6-item Likert scale measuring healthcare personnel’s professional perceptions regarding HCC surveillance, including perceived role importance, confidence in guideline knowledge, and team accountability; each item was rated on a 5-point scale (1 = Strongly Disagree/Not Important to 5 = Strongly Agree/Extremely Important), with a total possible score range of 6–30. Both instruments were developed by the principal investigator and reviewed for content validity by physician peers prior to use. Cronbach’s alpha values confirmed acceptable internal consistency for both instruments (Likert: α = 0.88 pretest, 0.90 posttest; Knowledge: α = 0.89 pretest, 0.86 posttest). Individual pairing of pre- and postintervention surveys was achieved through a unique anonymous identifier assigned to each participant at the outset of the study; participants recorded this identifier on both survey forms, enabling matched analysis while preserving confidentiality. Normality of knowledge score differences was verified using the Shapiro–Wilk test prior to applying the paired *t*-test. Paired *t*-test compared knowledge scores; Wilcoxon signed-rank test compared Likert-scale perception scores given their ordinal nature. An alpha level of 0.05 was used for all inferential tests. Statistical analyses were conducted using IBM SPSS Statistics (Version 28.0; IBM Corp., Armonk, NY, USA). No data were missing, as survey completion was required for inclusion. Surveillance process rates were compared descriptively between the preintervention and postintervention chart-review periods. Because data were collected as two discrete pre-post snapshots rather than as a longitudinal time series, run chart or statistical process control (SPC) chart analysis [12] was not feasible within this initial cycle. This constraint is acknowledged as a key limitation and is directly addressed in the Act phase recommendations below.

Act: Findings from this cycle establish the foundation for subsequent iterative PDSA cycles. The Act phase recommends: (1) implementation of run chart monitoring of surveillance ordering and scheduling rates monthly across the next four quarters to assess sustainment and identify special-cause variation; (2) role-specific educational reinforcement, with focused sessions for the scheduling and administrative staff who showed the largest coordination gaps; (3) integration of EHR-based population health reporting to proactively identify patients overdue for surveillance; and (4) expansion of the intervention to personnel not captured in this cycle.

### 2.4. Ethical Considerations

The study was conducted in accordance with the Declaration of Helsinki and was determined by the Institutional Review Board of Arkansas State University (FY24-25-77) not to constitute research involving human subjects. The IRB determined that the proposed activity does not meet the definition of ‘research involving human subjects’ as defined and codified at 45 CFR 46.102 by the US Department of Health and Human Services. The project posed minimal risk to participants. Participation was voluntary; informed consent was indicated by voluntary completion of the survey instruments. Participants were informed of their right to withdraw without penalty. All patient and personnel data were deidentified and stored in compliance with HIPAA regulations and institutional data security policies.

## 3. Results

### 3.1. Healthcare Personnel Demographics

Of 40 eligible healthcare personnel, 30 completed both pre- and postintervention surveys (75% participation). The sample was predominantly female (n = 27, 90.0%) and clinically experienced, with 56.7% reporting more than 10 years of ambulatory practice. Registered nurses comprised the largest role group (43.3%), followed by advanced practice providers, medical assistants, and support staff (13.3% each). Full demographics are presented in Table 1.

### 3.2. Patient Chart Review Demographics

Chart reviews included 50 preintervention and 50 postintervention patient records. The patient population was predominantly in the 51–70 age range (60% pre, 62% post) and was majority Caucasian (58% pre, 68% post), with African American patients representing the second-largest ethnic group (40% pre, 24% post). Gender distribution differed between the two review periods, with male patients comprising 56% of the preintervention cohort and female patients comprising 66% of the postintervention cohort, most likely reflecting variation in the composition of scheduled patients across review windows rather than a change in the target population. Full demographics are presented in Table 2.

### 3.3. Knowledge and Perception Outcomes

Preintervention knowledge scores revealed substantial baseline gaps: the mean score was 41.67% (SD = 15.33) of the total possible score, indicating limited familiarity with current HCC surveillance guidelines despite the team’s considerable clinical experience. Following the educational intervention, mean knowledge scores increased to 95.33% (SD = 8.60), representing a 53.66-percentage-point improvement. A two-tailed paired *t*-test confirmed this improvement was statistically significant (t [29] = −20.27, *p* < 0.001) with a very large effect size (Cohen’s d = 3.70), demonstrating the intervention’s substantial practical impact on knowledge acquisition.

Perception scores also improved significantly. Median Likert-scale scores increased from 21 on the pretest to 24 on the posttest, and the Wilcoxon signed-rank test confirmed a statistically significant difference (V = 4.00, z = −4.30, *p* < 0.001). Of note, for the item “How important is your role in supporting patient adherence to HCC surveillance?” (rated on a 5-point scale: 1 = Not important to 5 = Extremely important), the proportion of participants selecting “Extremely important” increased from 3% (1/30) preintervention to 93% (28/30) postintervention, signaling a meaningful shift in perceived professional accountability across the team. Statistical outcomes are summarized in Table 3.

### 3.4. HCC Surveillance Process Outcomes

Chart review data showed improvements in HCC surveillance process measures. Surveillance imaging ordering increased from 88% (44/50) preintervention to 94% (47/50) postintervention, an absolute difference of 6 percentage points (95% CI: −4% to 16%). Scheduling rates improved from 60% (30/50) to 80% (40/50), an absolute difference of 20 percentage points (95% CI: 5% to 35%), representing the most clinically meaningful process-level gain of this cycle. The ordering improvement was modest and the confidence interval crosses zero, indicating uncertainty in its magnitude; the scheduling improvement was larger and more precisely estimated. Preintervention data showed that 32% of ordered studies were completed and 28% of results were formally reviewed within the chart review window. Postintervention completion and review rates could not be captured within the project timeframe given the six-month recommended surveillance interval; ordering and scheduling rates therefore served as primary upstream process-level indicators. These data represent two discrete snapshots rather than a longitudinal time series; longitudinal run chart monitoring is planned for subsequent cycles. Surveillance rates are presented in Table 4.

### 3.5. Implementation Context

Implementation was facilitated by strong institutional support from the Advanced Liver Disease Center and transplant institute leadership, who reinforced the clinical urgency of early HCC detection in the context of liver transplantation as a curative treatment option. A concurrent transition to a new electronic health record system created challenges in retrospective data retrieval and in scheduling educational sessions within an active ambulatory workflow. Collaboration with clinic leadership and the clinic manager enabled the session to be delivered at a time that minimized disruption to patient care. Ongoing guidance from the project committee and practice partners supported the successful completion of the project within the planned timeline.

## 4. Discussion

This EBP-QI project found that a single, structured PDSA educational cycle was associated with significant improvements in healthcare personnel’s knowledge and perceptions regarding HCC surveillance and with clinically meaningful improvements in surveillance ordering and scheduling rates in an ambulatory hepatology setting. The magnitude of knowledge improvement, from a mean of 41.67% to 95.33%, and the very large effect size (Cohen’s d = 3.70) suggest that even a single, well-designed educational session can produce rapid and substantial gains in guideline knowledge across an experienced interdisciplinary team. These findings align with a growing body of evidence demonstrating that targeted educational interventions addressing provider knowledge deficits improve cancer surveillance utilization among high-risk populations and extend this evidence base to the specific context of ambulatory HCC surveillance [10].

The finding that baseline knowledge scores averaged only 41.67%, despite a team with substantial clinical experience, over half of whom had more than 10 years in ambulatory practice, warrants careful interpretation. It is unlikely that participants were unaware of HCC surveillance guidelines; rather, the questionnaire’s depth and specificity revealed gaps in understanding of the precise criteria, intervals, and modalities recommended by current AASLD guidelines. This distinction has important practical implications: clinicians who are broadly aware of the concept of HCC surveillance may nonetheless be uncertain about which patients qualify, what imaging modality is appropriate, or how frequently testing should occur, uncertainties that translate directly into noncompliance with HCC surveillance practices. Structured education that addresses these specifics, rather than general awareness campaigns, may be essential to closing the implementation gap.

The shift in perceived professional responsibility merits particular attention. The increase from only 3% (1/30) to 93% (28/30) of participants rating their own role in patient adherence as extremely important represents one of the most striking findings of this project. This baseline figure suggests that, prior to the intervention, most team members viewed HCC surveillance adherence as primarily the ordering clinician’s responsibility, with limited personal accountability for downstream processes such as scheduling, patient contact, and completion tracking. The educational intervention, which explicitly addressed the distinct surveillance and coordination roles of each team member, appears to have meaningfully reframed this perception, cultivating a shared sense of accountability across the team. In ambulatory settings where surveillance completion depends on coordinated action across multiple roles, this cultural shift may be at least as important as the knowledge gains themselves.

The 20 percent improvement in scheduling rates, from 60% to 80%, is the most clinically consequential process outcome of this cycle. Scheduling represents the critical operational handoff between a clinician’s ordering intent and a patient’s actual receipt of surveillance imaging. The persistence of a gap between ordering (94%) and scheduling (80%) following the intervention points to a residual system-level barrier that cannot be fully addressed through education alone. Likely contributors include challenges in reaching patients by phone, variable insurance authorization timelines, imaging appointment availability, and the absence of systematic electronic reminders or population health reporting tools to flag patients whose scheduled appointments were not confirmed. Identifying and addressing these structural barriers will be a primary focus of subsequent iterative PDSA cycles, supported by run chart monitoring to determine whether improvements are sustained or whether further testing of change is required.

The preintervention completion rate of 32% and result review rate of 28% are themselves clinically significant findings that warrant interpretive comment beyond their role as baseline comparators. These figures reveal that even when surveillance imaging was ordered, the first step in the process, the large majority of studies did not result in completed imaging or formally documented result review within the chart review window. This pattern is consistent with a well-described surveillance failure chain in which breakdown occurs not at the point of clinical intent but at the downstream operational steps of scheduling, patient follow-through, and result tracking. Addressing these structural and operational barriers will be a primary focus of subsequent PDSA cycles and will require interventions beyond education alone, such as automated tracking systems, patient navigation, and EHR-based population health alerts.

The absence of run chart or SPC chart data in this project is an acknowledged limitation of the current cycle. Because data were collected as two discrete pre- and postintervention snapshots rather than as a longitudinal time series across multiple measurement periods, it is not possible to determine from the current data whether the observed improvements reflect a true and sustained shift in surveillance process performance or a transient response to the intervention, including the possibility of a Hawthorne effect. This is a recognized constraint of single-cycle pre-post designs in QI practice, and is directly addressed in the Act phase recommendations, which establish monthly run chart monitoring of surveillance ordering and scheduling rates as the primary evaluation strategy for the next four quarters. This longitudinal approach will allow the team to distinguish between common-cause variation and special-cause signals, assess the durability of improvements, and identify the need for additional intervention cycles if performance regresses.

The Donabedian framework provided an effective and coherent structure for evaluating this project across three levels. At the structural level, the educational session, as an organized, guideline-based intervention delivered to the full interdisciplinary team, represented an investment in the care environment’s capacity to support quality surveillance. At the process level, improvements in ordering and scheduling rates demonstrated that structural changes translated into measurable changes in care delivery behaviors. At the outcome level, the knowledge and perception scores provide proximate evidence of the behavioral and attitudinal changes through which process improvements are mediated. Future cycles will extend this evaluation framework to distal outcome-level data, including surveillance completion rates, time from order to imaging, and, over a longer horizon, proportions of early-stage HCC detected among surveilled patients.

*NP Practice Implications*

These findings carry direct implications for interdisciplinary practice in hepatology and transplant settings. While nurse practitioners are optimally positioned to identify surveillance-eligible patients, place orders, and coordinate downstream processes, sustained improvement requires shared accountability across all team roles, including scheduling staff, medical assistants, and administrative personnel. The substantial baseline knowledge gap identified among experienced clinical staff underscores the need for recurring, structured education on HCC surveillance guidelines as an ongoing institutional commitment rather than a one-time initiative. Clinicians in QI leadership roles, regardless of discipline, can advance these goals by championing standardized surveillance protocols, team-based accountability structures, and EHR-based population health tools to proactively identify patients overdue for imaging.

*Limitations*

This project has several limitations that should be considered when interpreting findings and planning subsequent cycles. First, the single-site design within a specialized transplant hepatology clinic limits generalizability; the patient population, team composition, and institutional resources in this setting differ substantially from those in community-based hepatology practices and primary care settings, where the majority of patients with cirrhosis receive care. Replication across diverse clinical environments will be necessary to assess the broader applicability of the intervention.

Second, the pre-post design without a concurrent control group limits causal inference. Improvements in surveillance ordering and scheduling rates cannot be attributed exclusively to educational intervention, as concurrent factors, including the heightened institutional focus on quality metrics prompted by the EHR transition, increased leadership engagement during the project period, and natural variation in patient panel composition between the two chart review windows, may have independently influenced observed rates. A controlled design or stepped-wedge implementation across multiple clinic sites would provide stronger evidence of the intervention effect in future cycles.

Third, and most significantly for the EBP-QI design, surveillance data in this project were collected as two discrete pre- and postintervention snapshots rather than as a longitudinal time series. This precludes the use of run chart or SPC chart analysis, which are the gold-standard tools for distinguishing sustained improvement from transient response in QI evaluation [12]. Without serial data points across multiple measurement periods, it is not possible to determine whether the improvements in ordering and scheduling rates represent a durable shift in process performance or a Hawthorne effect that may attenuate as the intervention recedes. Prospective run chart monitoring across four subsequent quarters has been designated as the primary evaluation strategy for the next PDSA cycle, with the specific intent of generating the longitudinal data necessary to make this determination.

Fourth, postintervention surveillance completion and result review rates could not be captured within the project timeframe, given the six-month recommended surveillance interval. Ordering and scheduling rates, therefore, served as proxies for adherence, capturing the upstream process rather than the downstream outcome of completed imaging. Whether the process improvements observed translate into higher rates of timely surveillance completion among eligible patients will be assessed in subsequent cycles with extended follow-up windows.

Fifth, not all eligible healthcare personnel participated, as 10 of 40 team members were absent on the day of the educational session due to clinical scheduling constraints. Of the absent personnel, the majority were schedulers and administrative support staff, the roles with the greatest downstream impact on surveillance completion. This introduces potential selection bias, as participants may have had a greater pre-existing interest in quality improvement or surveillance practices, and may also explain the residual ordering-to-scheduling gap observed postintervention. Absent personnel represent a priority group for inclusion in subsequent cycles, with flexible delivery options, including recorded sessions and small-group make-up sessions, considered to maximize reach.

Sixth, eligibility adjudication for hepatitis B patients was performed by the principal investigator through chart review against AASLD risk criteria and was not independently verified, representing a potential source of denominator inaccuracy. Because AASLD surveillance criteria for hepatitis B are nuanced and depend on multiple patient-level factors, unverified adjudication introduces the possibility that some patients included in the denominator may not have met full eligibility thresholds. Future cycles should incorporate a structured eligibility checklist verified by a second clinician to ensure that the surveillance-eligible population is consistently and accurately defined.

Finally, the knowledge and Likert-scale instruments used in this project were developed by the principal investigator and reviewed for content validity by physician peers; they have not undergone formal psychometric validation in external populations. The Cronbach’s alpha values indicating acceptable internal consistency provide some assurance of instrument reliability, but established, externally validated instruments should be considered for use in future cycles to strengthen the measurement framework. The substantial improvement in knowledge scores (from 41.67% to 95.33%) warrants careful interpretation. The postintervention knowledge assessment was administered immediately following the educational session. Consequently, the posttest scores may reflect short-term recall, repeated exposure to similar item formats, or social desirability bias rather than durable knowledge retention. Future cycles should include a delayed posttest (e.g., 30 to 90 days following the intervention) to evaluate sustained knowledge acquisition. Additionally, this project did not prospectively measure balancing measures. Potential unintended consequences of the intervention, including increased workload for nursing and scheduling staff, delays in other clinic tasks, duplicate imaging orders, insurance authorization delays, or patient cancellations and no-shows, were not assessed. The absence of balancing measures should be acknowledged as a limitation of the current cycle, and their prospective collection is planned for subsequent PDSA cycles.

## 5. Conclusions

This EBP-QI project found that one completed PDSA cycle, centered on a structured, guideline-based educational intervention delivered to an interdisciplinary ambulatory hepatology team, was associated with statistically significant improvements in HCC surveillance knowledge and professional perceptions, as well as improved process-level adherence indicators. The large effect size observed for knowledge acquisition, the striking shift in perceived team accountability, and the 20-percentage-point improvement in surveillance scheduling rates collectively support structured team education as a feasible, evidence-based QI strategy to address HCC surveillance gaps in ambulatory practice, even within the constraints of a single improvement cycle. However, imaging completion and result-review outcomes, sustainability, and causal attribution require evaluation through longitudinal monitoring and subsequent PDSA cycles.

At the same time, the limitations of this initial cycle are important to acknowledge candidly. The pre-post snapshot design does not provide the longitudinal data needed to determine whether the observed improvements are sustained over time or represent a transient response to the intervention. The absence of run chart or SPC chart monitoring is the most substantive methodological gap in the current cycle and must be rectified in subsequent work. The next PDSA cycle will prioritize monthly run chart tracking, surveillance, ordering, and scheduling rates over a minimum of four consecutive quarters, creating the time-series data necessary to apply standard QI analytical methods and to distinguish durable process improvement from common-cause variation.

Beyond run chart implementation, sustained improvement will require iterative testing of additional changes across subsequent PDSA cycles. These include integrating EHR-based population health reporting to proactively identify patients overdue for imaging, developing standardized workflows for patient outreach and follow-through scheduling, and expanding the educational intervention to personnel not reached in the initial cycle. Addressing the residual ordering-to-scheduling gap, which persisted at 14 percentage points after the intervention, will require not only educational reinforcement but also structural changes to the scheduling and patient-communication infrastructure that cannot be fully addressed by knowledge improvement alone.

Ultimately, improving HCC surveillance adherence at the population level will require coordinated action across multiple clinical settings, institutional leadership commitment to quality monitoring, and interdisciplinary team cultures that distribute accountability for surveillance completion across all team roles. This project demonstrates that meaningful progress toward that goal is achievable through focused, evidence-based QI efforts and that nurse practitioners, as central coordinators of chronic liver disease management, are well-positioned to lead the iterative improvement cycles needed to sustain and build on these early gains.

## Figures and Tables

**Table 1:** Demographic Characteristics of Healthcare Personnel (N = 30).

Characteristic	n	Percent (%)
Gender		
Female	27	90.0
Male	3	10.0
Prefer not to respond	0	0.0
Clinical Role		
Registered Nurse	13	43.3
Advanced Practice Provider	4	13.3
Medical Assistant	4	13.3
Support Staff	4	13.3
Physician	2	6.7
Scheduler	2	6.7
Student	1	3.4
Years of Clinical Experience		
>10 years	17	56.7
0–5 years	9	30.0
6–10 years	4	13.3

**Table 2:** Demographic Characteristics of Patients Included in Chart Review (Pre n = 50; Post n = 50).

Characteristic	Pre n	Post n	Pre %	Post %
Age (years)				
20–35	2	0	4.0	0.0
36–50	8	11	16.0	22.0
51–70	30	31	60.0	62.0
>70	10	8	20.0	16.0
Gender				
Male	28	17	56.0	34.0
Female	22	33	44.0	66.0
Not recorded	0	0	0.0	0.0
Ethnicity				
Caucasian	29	34	58.0	68.0
African American	20	12	40.0	24.0
Hispanic	1	1	2.0	2.0
Asian	0	3	0.0	6.0

Note. Ethnicity categories reflect patient self-report as recorded in the electronic health record.

**Table 3:** Pre- and Postintervention Knowledge and Perception Outcomes (N = 30).

Outcome	Pre M (SD)	Post M (SD)	Statistic	*p*	Effect Size	Test
Knowledge Score	41.67 (15.33)	95.33 (8.60)	t(29) = −20.27	<0.001	d = 3.70	Paired *t*-test
Perception (Likert)	Mdn = 21	Mdn = 24	z = −4.30	<0.001	—	Wilcoxon

Note. Knowledge instrument: 15-item multiple-choice questionnaire; scores expressed as percentage of total possible score (range 0–100%). Perception instrument: 6-item Likert scale (each item rated 1–5); total score range 6–30; scores expressed as median. d = Cohen’s d effect size for paired *t*-test.

**Table 4:** HCC Surveillance Process Rates Pre- and Postintervention.

Surveillance Indicator	Pre-Intervention n (%)	Post-Intervention n (%)	Absolute Difference (95% CI)
Imaging ordered	44/50 (88%)	47/50 (94%)	+6 pp (−4% to 16%)
Imaging scheduled	30/50 (60%)	40/50 (80%)	+20 pp (5% to 35%)
Imaging completed	16/50 (32%)	N/A ^†^	-
Results reviewed	14/50 (28%)	N/A ^†^	-

^†^ Postintervention completion and review rates were not assessable within the project timeframe due to the six-month recommended surveillance interval. Completion rate tracking is a primary objective of subsequent PDSA cycles.

## Data Availability

All datasets collected for this project are presented within the manuscript. Any further queries can be directed to the corresponding author.

## Public Involvement Statement

No public involvement in any aspect of this research.

## Guidelines and Standards Statement

The clinical content of this manuscript was based on guidance from the American Association for the Study of Liver Diseases (AASLD) [7]. Reporting was guided by the SQUIRE 2.0 (Standards for QUality Improvement Reporting Excellence) guidelines for quality improvement reporting.

## Abbreviations

The following abbreviations are used in this manuscript:

HCC	Hepatocellular carcinoma
PDSA	Plan-Do-Study-Act
AASLD	American Association for the Study of Liver Diseases
QI	Quality improvement
EBP	Evidence-based practice
AFP	Alpha-fetoprotein
EHR	Electronic health record
IRB	Institutional Review Board
NP	Nurse practitioner
APP	Advanced Practice Provider
RN	Registered Nurse
MA	Medical Assistant
SPC	Statistical process control
HIPAA	Health Insurance Portability and Accountability Act
SD	Standard deviation
